# Solvothermal Engineering of NaTi_2_(PO_4_)_3_ Nanomorphology for Applications in Aqueous Na-Ion Batteries

**DOI:** 10.1021/acssuschemeng.2c06732

**Published:** 2023-02-15

**Authors:** Gintarė Gečė, Jurgis Pilipavičius, Nadežda Traškina, Audrius Drabavičius, Linas Vilčiauskas

**Affiliations:** Center for Physical Sciences and Technology (FTMC), Saulėtekio al. 3, LT-10257 Vilnius, Lithuania

**Keywords:** Sodium titanium phosphate, NASICON, Hydro(solvo)thermal synthesis, Aqueous sodium-ion batteries, Morphology control, Water activity

## Abstract

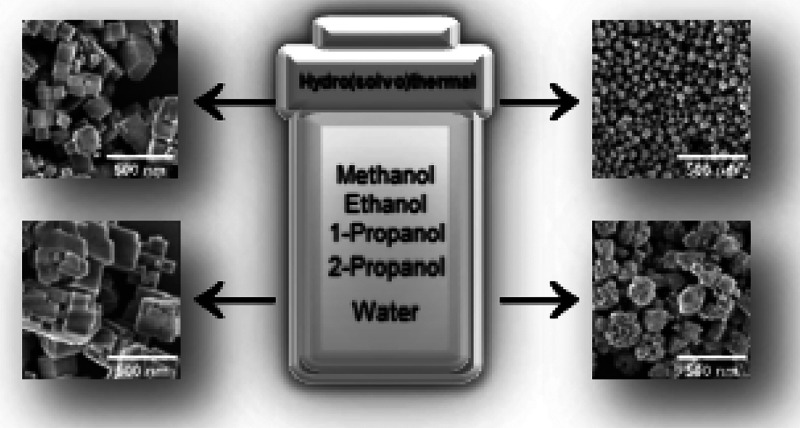

Aqueous Na-ion batteries are among the most discussed alternatives to the currently dominating Li-ion battery technology, in the area of stationary storage systems because of their sustainability, safety, stability, and environmental friendliness. The electrochemical properties such as ion insertion kinetics, practical capacity, cycling stability, or Coulombic efficiency are strongly dependent on the structure, morphology, and purity of an electrode material. The selection and optimization of materials synthesis route in many cases allows researchers to engineer materials with desired properties. In this work, we present a comprehensive study on size- and shape-controlled hydro(solvo)thermal synthesis of NaTi_2_(PO_4_)_3_ nanoparticles. The effects of different alcohol/water synthesis media on nanoparticle phase purity, morphology, and size distribution are analyzed. Water activity in the synthesis media of different alcohol solutions is identified as the key parameter governing the nanoparticle phase purity, size, and shape. The careful engineering of NaTi_2_(PO_4_)_3_ nanoparticle morphology allows control of the electrochemical performance and degradation of these materials as aqueous Na-ion battery electrodes.

## Abstract 2

Comprehensive study on size- and shape-controlled hydro(solvo)thermal synthesis of NaTi_2_(PO_4_)_3_ for applications in aqueous Na-ion batteries.

## Introduction

Electrochemical technologies have a number of advantages over other means of storing electrical energy which include a wide range of available energy and power, high efficiency, facile scalability, low maintenance, or an easy integration into the grid.^1,2^ Aqueous Na-ion batteries (ASIBs) based on abundant electrode materials and simple electrolyte solutions are among the most sought after alternatives to the currently dominating Li-ion battery technology. They are especially suitable for stationary storage systems because of their sustainability, safety, stability, and environmental friendliness.^3−6^

The search and development of suitable electrode materials remains one of the major challenges in ASIB research. Various polyanionic or mixed-polyanion compounds such as NASICON-type materials stand out due to their structural and thermal stability, tunable redox potentials, low cost, and environmental friendliness.^7^ NASICON structured NaTi_2_(PO_4_)_3_ (NTP), with its favorable potential at ∼−0.6 V vs SHE (∼2.1 V vs Na^+^/Na) and excellent rate capability for reversible intercalation of two Na-ions per formula unit (theoretical capacity 133 mAh g^–1^), is an excellent ASIB anode candidate.^8^

The electrochemical properties such as ion insertion kinetics, practical capacity, cycling stability, or Coulombic efficiency are strongly dependent on the structure, morphology, and purity of electrode materials. The selection and optimization of materials synthesis route in many cases allow the engineering of materials with desired properties. This not only involves the preparation of primary powder but also its after-treatment by chemical, thermal, and physical means. Solid-state prepared NTP shows good electrochemical performance such as rate capability and cycling stability.^9^ However, poor particle size and morphology control limit the potential of this method for fine-tuning the nanoscale morphology. On the other hand, sol–gel synthesis is another simple, scalable, and widely used method. Various aspects of the sol–gel technique for preparing NTP on the material purity, degree of crystallinity, and properties of carbonaceous phase were recently investigated.^10^ Hydro(solvo)thermal synthesis is widely regarded as one of the most suitable soft chemical methods for preparing battery materials yielding high phase purity and crystallinity, narrow particle size distribution, and the ability to finely control particle morphology and agglomeration.^11^ All of this allows the design of particles with shorter ionic and electronic transporting paths leading to better electrochemical and charge storage properties. NTP particles with diverse morphologies such as cubic^12^ and plate-like morphologies,^13^ mesoporous microflowers,^14^ and nanowire clusters,^15^ etc. were reported. Small particles yield a large surface area and faster Na-ion insertion kinetics, and certain particle shapes such as cubic or spherical could result in special packings with lower steric hindrance and better contact.^16^ It is well-known that, among many factors, which govern crystal shape such as nucleation rate, temperature, pressure, precursor concentration, directing agents, etc., it is the solvothermal synthesis reaction medium that plays the major role in phase formation and nucleation kinetics controlling the size and morphology of the resulting nanoparticles.^17^ However, a clear understanding between the hydro(solvo)thermal synthesis conditions and the resulting morphology is still lacking. Although various organic solvents are widely used in solvothermal synthesis, some of them are expensive, toxic, and environmentally harmful.^18^ Therefore, the potential to use water or aqueous mixtures is highly desirable due to their solvating power and sustainability.^19,20^

In this work, we present a comprehensive study on size- and shape-controlled hydro(solvo)thermal synthesis of NTP. We analyze the effects of different alcohol/water reaction media on nanoparticle phase purity, morphology, and size distribution. The studied alcohols are methanol, ethanol, 1-propanol, 2-propanol, and ethylene glycol. The electrochemical properties of obtained materials as ASIB negative electrodes are analyzed by voltammetric and galvanostatic cycling methods. Water activity in hydro(solvo)thermal synthesis medium is identified as the key parameter governing the phase purity and morphology of NTP. Different studied alcohols show significantly different water binding abilities and hydro(solvo)thermal synthesis yields. On the basis of obtained results, a viable NTP synthesis strategy for preparing the most suitable NTP particles for applications in ASIBs is suggested.

## Experimental Section

### Synthesis

**Figure 1:**
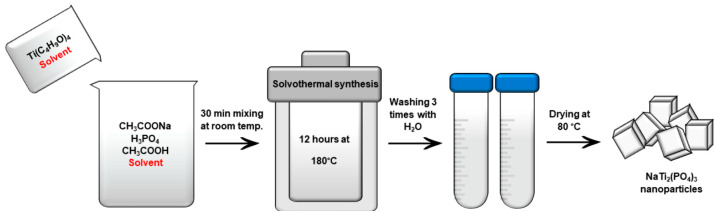
Solvothermal synthesis scheme of NaTi_2_(PO_4_)_3_.

NaTi_2_(PO_4_)_3_ nanoparticles were synthesized by a solvothermal method (Figure 1). In a typical synthesis, 0.246 g of CH_3_COONa (Chempur, ≥99.0%) was dissolved in 10 mL of H_3_PO_4_ (Reachem, 85 wt %), and then 10 mL of CH_3_COOH (Lach-ner, 99.8%) together with 40 mL of solvent were added to the mixture. The solvent consisted of one of the alcohols (methanol (CH_3_OH, Reachem), ethanol (CH_3_CH_2_OH, Honeywell), 1-propanol (CH_3_CH_2_CH_2_OH, Chempur), 2-propanol (CH_3_CHOHCH_3_, Reachem), or ethylene glycol (CH_2_OHCH_2_OH, Chempur)) and deionized water in different volume ratios (5:0, 4:1, 3:2, and 2:3). Afterward, a separate mixture of 1.4 mL titanium(IV) butoxide (C_16_H_36_O_4_Ti, Acros Organics, ≥98%) and 10 mL of solvent was prepared and then dropwise added into the previous solution under magnetic stirring. The final solution obtained after continuous stirring for 30 min at room temperature was transferred into a 100 mL Teflon-lined stainless-steel autoclave and heated at 180 °C for 12 h. Eventually, the obtained white precipitate was collected, washed several times by centrifugation with distilled water, and later dried at 80 °C overnight. The resulting particles were coated with a layer of carbon by homogeneously mixing 0.70 g of NTP powder and 0.30 g of citric acid (HOC(CH_2_CO_2_H)_2_, Lach-ner, G.R.) in 50 mL of distilled water. The resulting mixture was dried at 80 °C for complete water elimination. The obtained white powder was reground and heated at 700 °C for 2 h in the N_2_ atmosphere.

### Materials Characterization

Powder X-ray diffraction (XRD) patterns were recorded on a Rigaku MiniFlex II diffractometer within the range 10°  ≤  2θ ≤  70° using Ni-filtered Cu K_α_ radiation. The scanning speed and step width were 3° min^–1^ and 0.02°, respectively. The morphological and size characterization was carried out using a Helios Nanolab 650, FEI scanning electron microscope (SEM), and Tecnai G2 F20 X-TWIN, FEI transmission electron microscope (TEM). ImageJ software^21^ was used for particle size distribution determination. Powder surface area was measured by N_2_ adsorption isotherms at 77 K using the Anton Paar Brunauer–Emmett–Teller (BET) analyzer. Before the gas sorption measurements, all analyzed powder samples were outgassed in the vacuum at 90 °C for 3 h. Total surface area was estimated by the Brunauer–Emmett–Teller model. Thermogravimetric analysis (TGA) for the determination of carbon content was carried out on a STA600 PerkinElmer analyzer in the range of 30 to 700 °C at a heating rate of 20 °C min^–1^ in air atmosphere (20 mL min^–1^).

### Electrochemical Characterization

The electrode slurry was prepared by mixing 70 wt % of active material, 20 wt % of carbon black (CB) (Super-P, TIMCAL), and 10 wt % of polyvinylidene fluoride (PVDF) (HSV1800, Kynar) in *N*-methyl-2-pyrrolidone (NMP) (Sigma-Aldrich, 99.5%). The slurry was homogenized in a high-energy ball-mill (1 h at 175 rpm and 2 h at 350 rpm) using 5 mm ZrO_2_ balls at the ball-to-sample ratio of 3:1, then casted as a film and dried in vacuum for 3 h at 120 °C. The resulting electrode film was pressed on 316L stainless steel (SS) mesh (#325) and punched into disks with an average active material loading of ∼2.5 mg cm^–2^ (∼0.333 mAh cm^–2^). Electrochemical properties of the electrodes were characterized in a three-electrode bottom-mount beaker-type cell designed for flat samples using Na_2_SO_4_ (aq.) (10 mL, 1 M) electrolyte solution, and Ag/AgCl/3 M KCl reference and graphite-rod (60 mm in length and 5 mm in diameter) counter electrodes, respectively. All voltammetric measurements were performed on a PGSTAT-302 Metrohm Autolab potentiostat-galvanostat. The galvanostatic charge/discharge cycling was carried out on a Neware CT-4008–5 V10 mA cycler.

## Results and Discussion

### Structural Analysis

**Figure 2:**
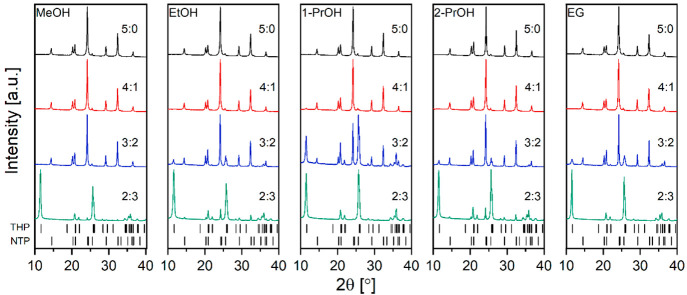
Powder XRD patterns of NTP samples prepared in different solvothermal synthesis media (5:0, 4:1, 3:2, and 2:3 correspond to volume ratios between water and alcohol).

A series of NTP nanoparticle samples were prepared by a solvothermal method using different synthesis media. Five different alcohols such as methanol (MeOH), ethanol (EtOH), 1-propanol (1-PrOH), 2-propanol (2-PrOH), and ethylene glycol (EG) were selected and used either pure (denoted as 5:0 ratio) or mixed with water at different volume ratios: 4:1, 3:2, and 2:3. The obtained powder XRD patterns presented in Figure 2 show that in the case of pure solvents the syntheses always yield a pure NASICON-type NTP phase. The observed sharp diffraction peaks indicate a high degree of crystallinity and agree very well with the standard PDF card (PDF#96-153-0650) indexed to the *R*3*c* (No. 167) space group.

Small additional peaks could be observed at 11.5, 25.7, 25.9° only for 4:1 1-PrOH, 3:2 EtOH, 2-PrOH and EG, and 2:3 MeOH systems. In all cases, these could be attributed to the monoclinic α-Ti(HPO_4_)_2_·H_2_O (THP) (PDF#96-100-6112) phase (*P*2_1_/*c* space group). The amount of THP increases with increasing water concentration in reaction media. This is a result of immediate hydrolysis and polycondensation of titanium butoxide to TiO_2_ in the presence of water also witnessed by the appearance of white milky suspension which under hydro(solvo)thermal conditions further reacts with phosphoric acid to yield THP.

**Figure 3:**
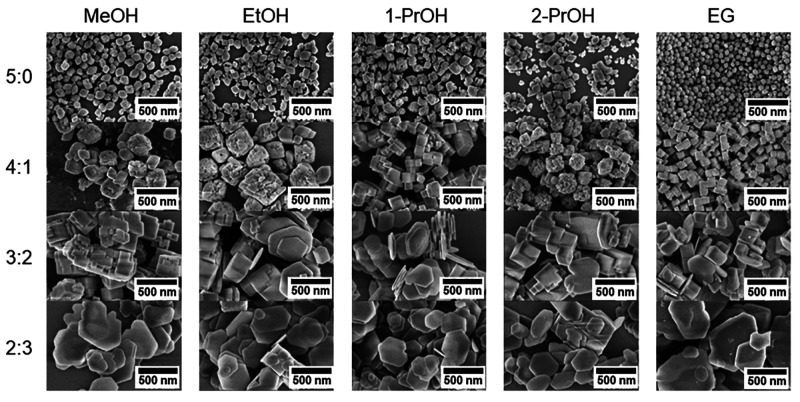
SEM micrographs of NTP sample nanomorphology prepared in different solvothermal synthesis media.

**Figure 4:**
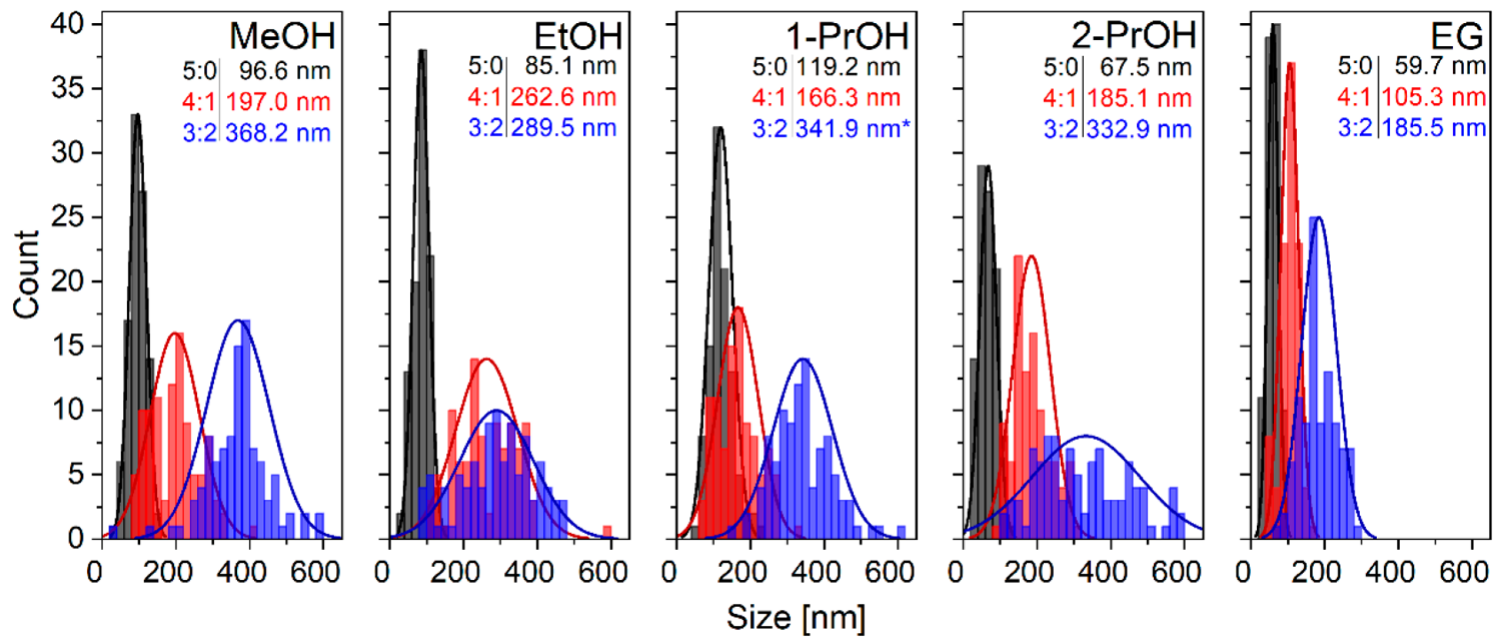
Particle size distribution histograms of NTP samples prepared in different solvothermal synthesis media as determined by ImageJ software^21^ (asterisk (*) denotes that the particles are mostly comprised of impurity phase).

The effect of reaction medium is also observed in the NTP particle shape and size distribution. Different morphologies as observed by SEM are presented in Figure 3. The particle size distribution analyses from SEM micrographs are presented in Figure 4. Nanoparticles obtained from pure alcohols have cubic-like morphology except those resulting from EG, which have a more spherical shape. The results show that the smallest particles (∼58 nm) are obtained from media with stronger intermolecular interactions and high viscosity which limits the diffusion and prevents particles from growing^22^ whereas the addition of water leads to increasing particle size and changes in shape. In the case of MeOH, the addition of water leads to defects appearing on the surface of cubic particles while for EtOH and 2-PrOH, the particles become irregular with a lot of defects resembling agglomerates. However, this is not observed for 1-PrOH and EG, where nanoparticles look regular cubic and are very uniform in size. If the MeOH to water ratio is 3:2, the particles become sharper with irregular crystals growing on top of each other which is not observed in other cases. An additional THP impurity phase could be also clearly observed due to its specific hexagonal microplate morphology.^23^

**Figure 5:**
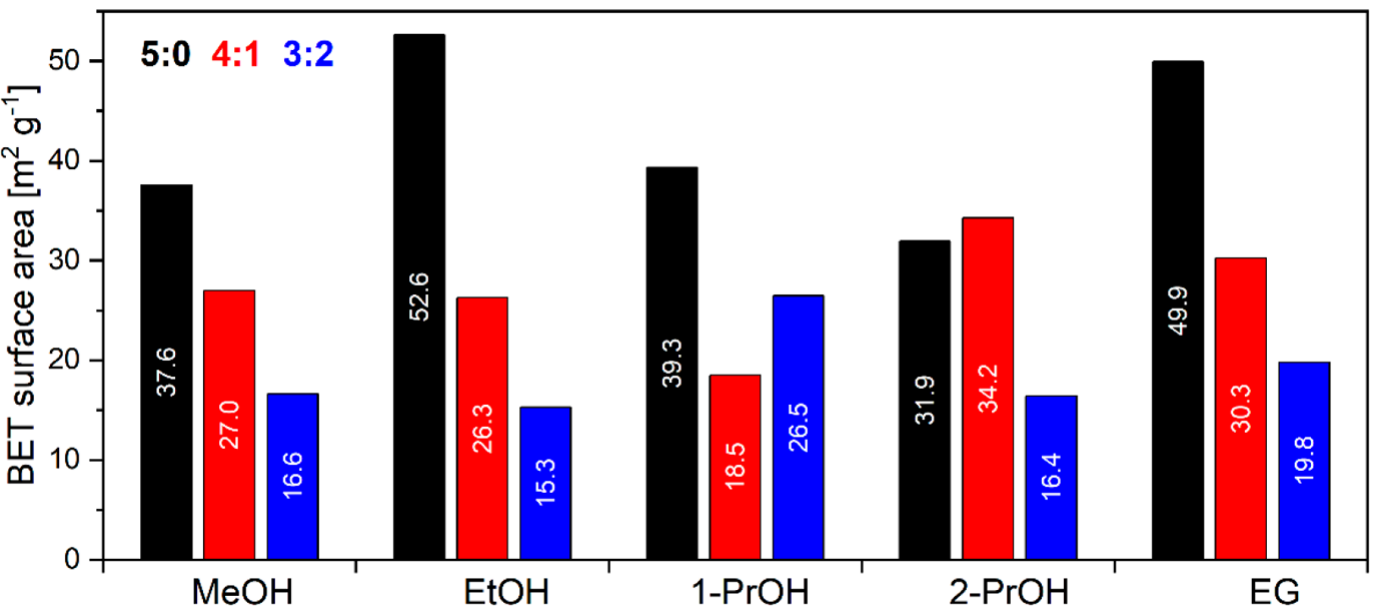
BET surface area analysis results for NTP samples prepared in different solvothermal synthesis media.

The results of BET surface area estimation are presented in Figure 5. The results show a clear correlation between the measured specific surface area and the nanoparticle size and morphology. Smaller nanoparticles obtained from pure alcohols have higher surface area (31.9 to 52.6 m^2^ g^–1^) than those obtained with an addition of water (15.3–34.2 m^2^ g^–1^). This is a direct result of increasing irregularity in particle shape and surface.

**Figure 6:**
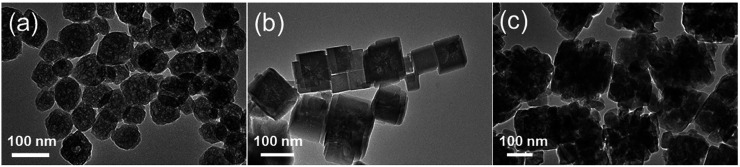
TEM images of NTP samples prepared in different solvothermal synthesis media: (a) pure EG, (b) 4:1 EG:water, (c) 4:1 2-PrOH:water.

TEM was carried out on some representative samples in order to investigate the particle morphology in more detail. Figure 6 shows TEM images of three different morphologies: spherical (5:0 EG:water), cubic (4:1 EG:water), and irregular (4:1 2-PrOH:water). It is obvious that spherical and irregular particles are composed from smaller NTP crystallites whereas cubic particles resemble small monocrystals. This suggests that different nanoparticles went through different particle formation and growth mechanisms. Results suggest that the spherical and irregular particles have been formed by coalescent growth while cubic ones are formed by Ostwald ripening.^24^ Moreover, dissimilarity in the morphology of spherical and irregular particles can be related to the different surface tensions of ethylene glycol and 2-propanol.^25,26^

**Figure 7:**
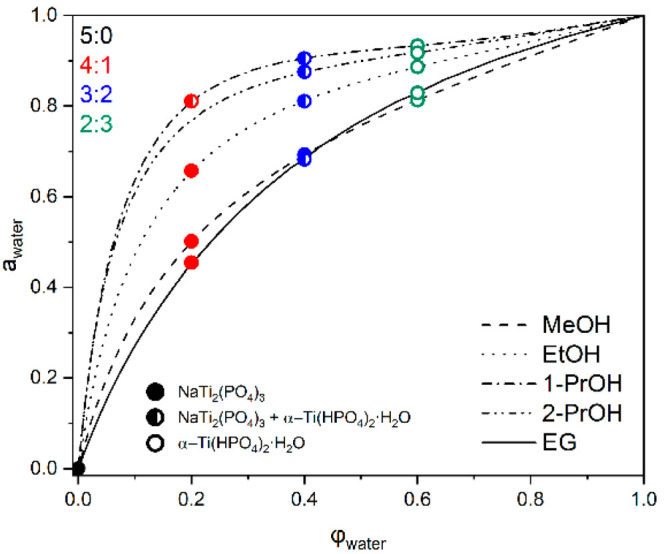
Relative water activity in different reaction media used in this work as evaluated by the AIOMFAC model.^27−29^

The structural and morphological analyses show that different synthesis media containing various alcohols and water contents result in significantly different NTP nanoparticles with no obvious correlation in terms of alkyl chain length, branching, or number of hydroxyl groups. One is clear: increasing water content is detrimental to NTP purity and limits the ability to control particle morphology. The results indicate that different alcohols have different interactions with water and the ability to bind it. For this reason, we indicated relative water activity (*a*_W_) in the solvothermal reaction medium as an effective parameter to quantify the inermolecular interactions able to characterize and explain the NTP solvothermal synthesis results. For this purpose, we use a semiempirical Aerosol Inorganic–Organic Mixtures Functional groups Activity Coefficients (AIOMFAC) thermodynamic model designed for the calculation of activity coefficients of different chemical species in inorganic–organic mixtures.^27−29^ Although the relative activities are estimated at ambient conditions whereas the true system in the solvothermal reactor is much more complex due to the presence of multiple salts of varying solubility, the results show that *a*_W_ could serve as a very good proxy parameter to characterize synthesis. The results of relative water activity with respect to medium composition as a function of volume fraction (φ_water_) and solvothermal synthesis yield are presented in Figure 7. They indicate positive mixing enthalpy for all alcohols with 1-PrOH deviating the most from ideality and EG the least. Therefore, this shows 1-PrOH to be poorly interacting with water which retains the highest activity among studied mixtures. The results clearly indicate that all systems where relative water activity exceeds ∼0.65 lead to either a significant presence of THP or no NTP phase at all. This relation could serve as an important guide for understanding the solvothermal synthesis conditions in terms of medium composition not only for NTP but also, potentially, for many other systems.

**Figure 8:**
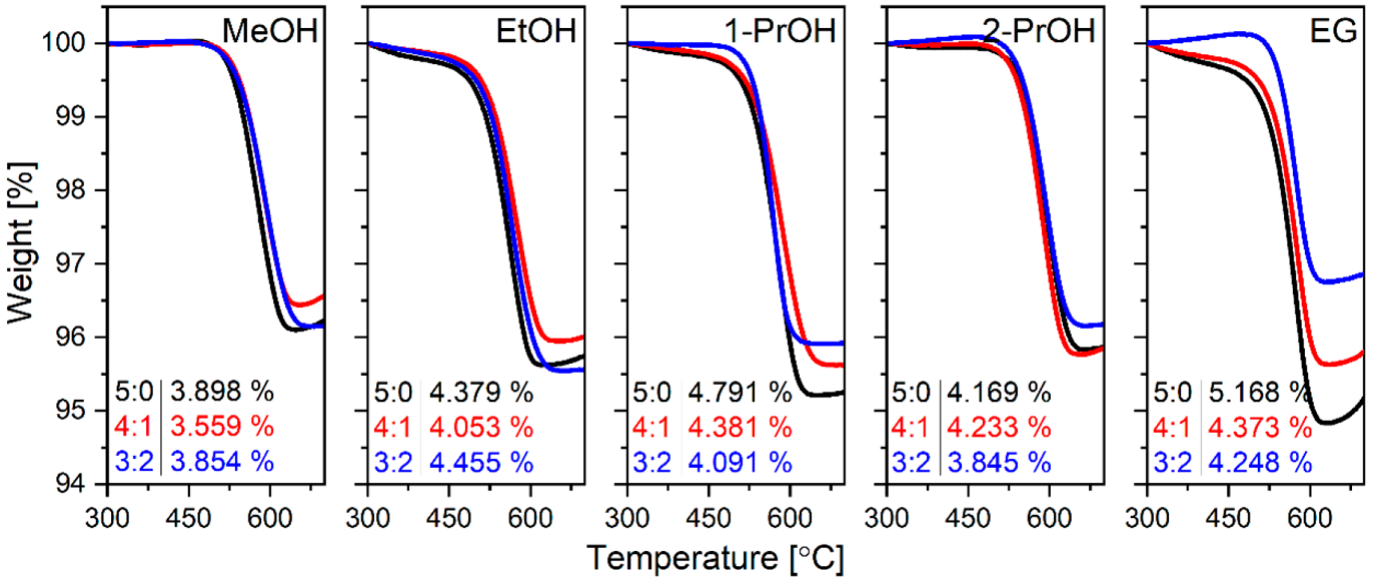
Thermogravimetric curves of NTP samples prepared in different solvothermal media.

In order to improve the electrochemical properties and the electronic contact between NTP nanoparticles, they were coated with a carbon layer by pyrolysis of citric acid at 700 °C. Phase morphology and size do not appear to be altered by the additional treatment.^30^ From the thermogravimetric results which are represented in Figure 8 one could see that all samples have a similar amount of about 4 wt % carbon irrespective of solvothermal synthesis conditions.

### Electrochemical Analysis

**Figure 9:**
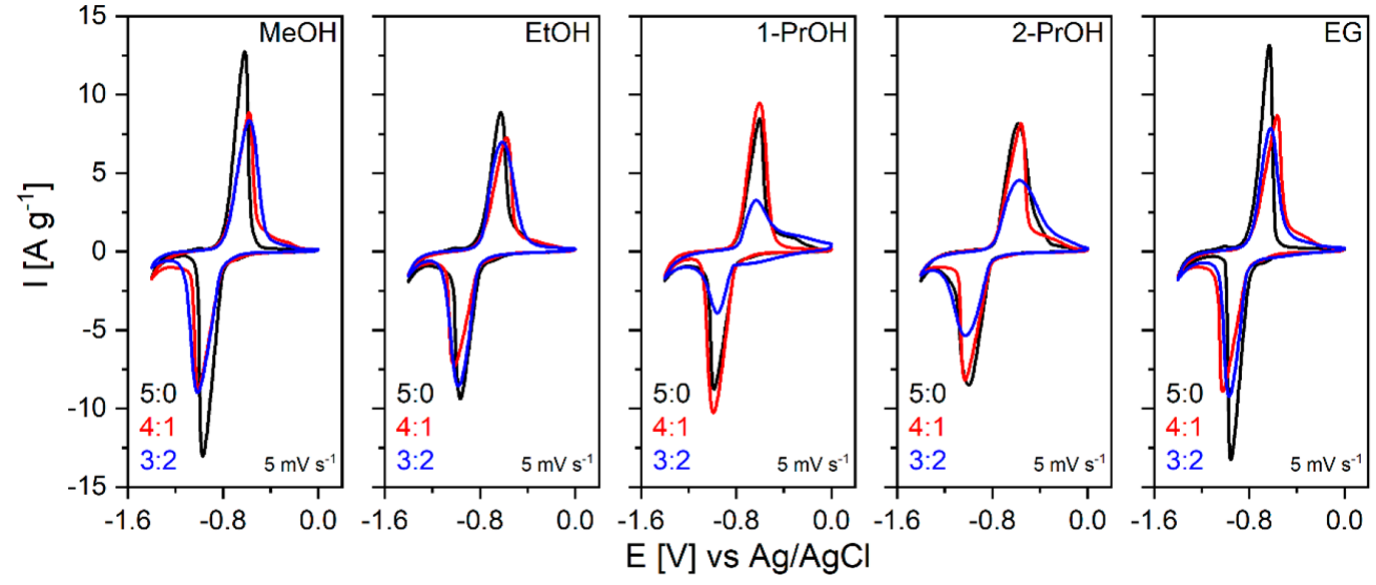
Cyclic voltammograms of NTP samples prepared in different solvothermal media (2nd CV cycle) recorded at 5 mV s^–1^ scan rate.

Electrodes for electrochemical characterization were formed from those samples which yielded mostly pure phase NTP. The electrochemical performance was evaluated using a three-electrode beaker-type bottom-mount cell specially designed for flat samples. Figure 9 shows the cyclic voltammograms (CV) of NTP electrodes recorded in aqueous solutions of 1 M Na_2_SO_4_ at a scanning rate of 5 mV s^–1^ within the potential window from −1.4 to 0 V vs Ag/AgCl. All samples have reduction and oxidation peaks at around −1.0 V and −0.6 V, respectively, which correspond to a reversible Ti^4+^/Ti^3+^ redox transition accompanied by insertion/deinsertion of Na^+^ ions: NaTi_2_(PO_4_)_3_ + 2Na^+^ + 2e^–^ ↔ Na_3_Ti_2_(PO_4_)_3_. The current peaks of the NTP synthesized either from pure MeOH or EG are higher and sharper with smaller separation in terms of potential values. This could be attributed to a higher specific surface area, fewer surface defects, better contact with the conductive carbon, and a more close packed structure of a composite. The wider and lower peaks of NTP samples obtained from EtOH or 2-PrOH could be explained by more irregular particle shapes and broader particle size distribution affecting the electrochemical kinetics. It should be noted that the amount of impurity phase was not evaluated explicitly; therefore, some drop in measured specific currents for certain samples could be due to electrochemically inactive THP.

**Figure 10:**
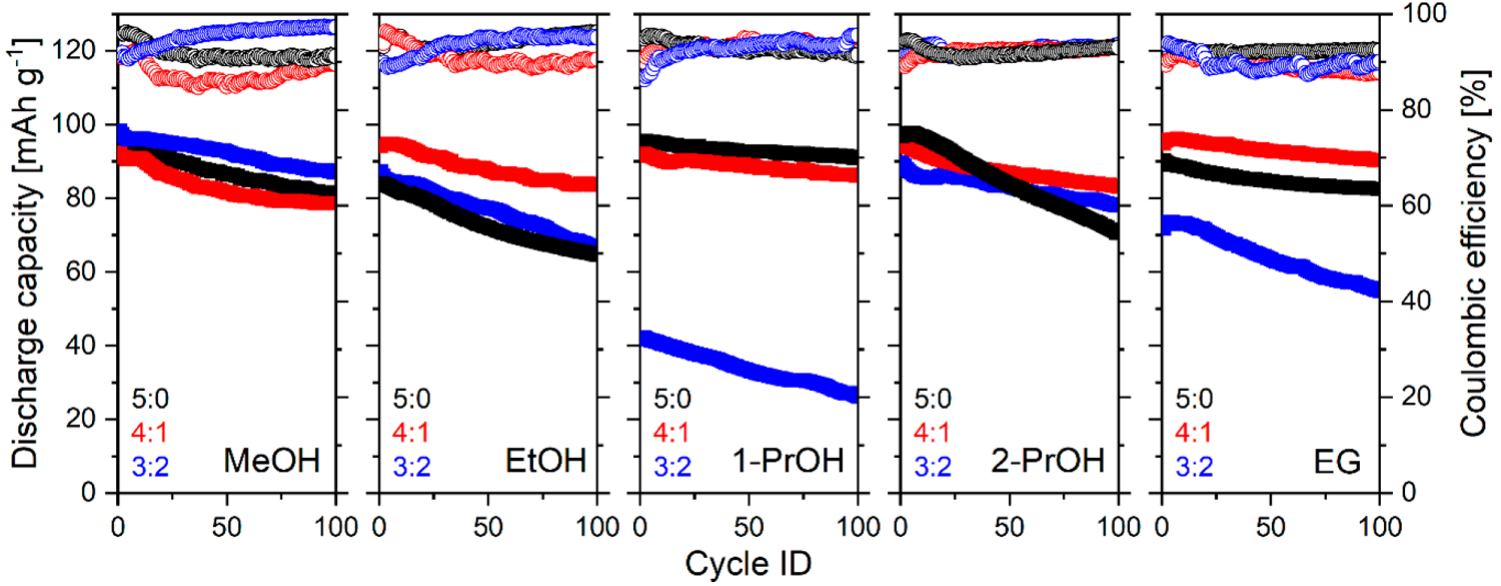
Galvanostatic cycling performance of NTP prepared in different solvothermal media in 1 M Na_2_SO_4_ (aq) solution at 1C rate.

**Figure 11:**
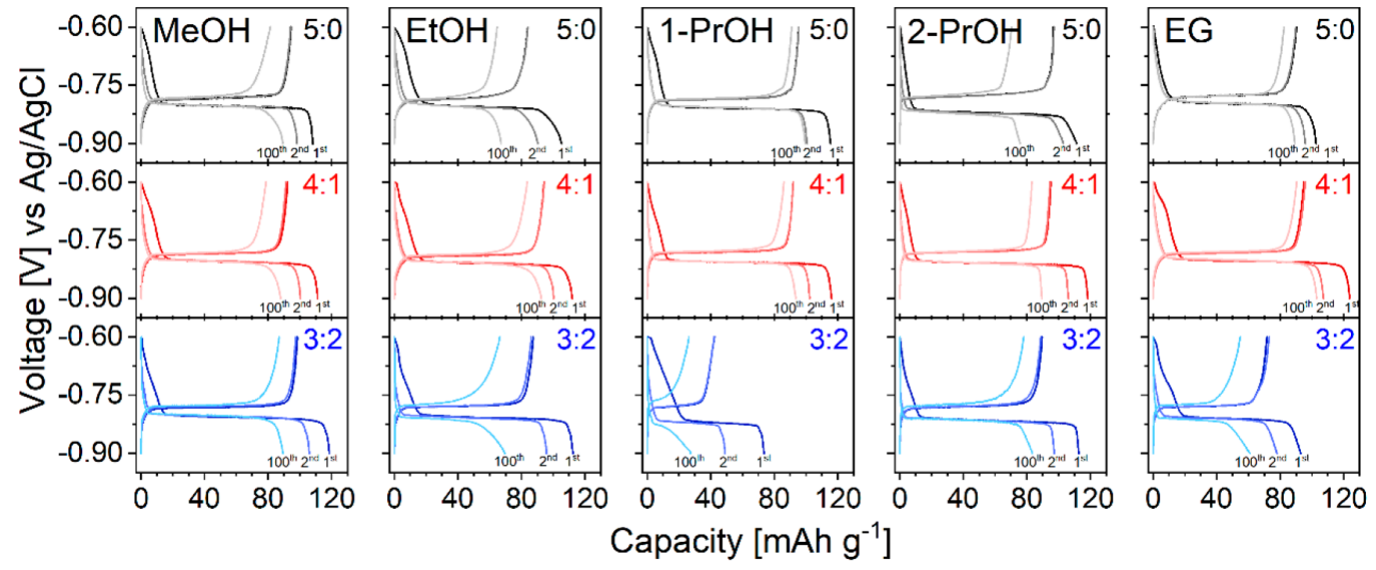
Galvanostatic charge–discharge curves for the first, second, and 100th cycles of NTP prepared in different solvothermal media in 1 M Na_2_SO_4_ (aq) solution at 1C rate.

**Figure 12:**
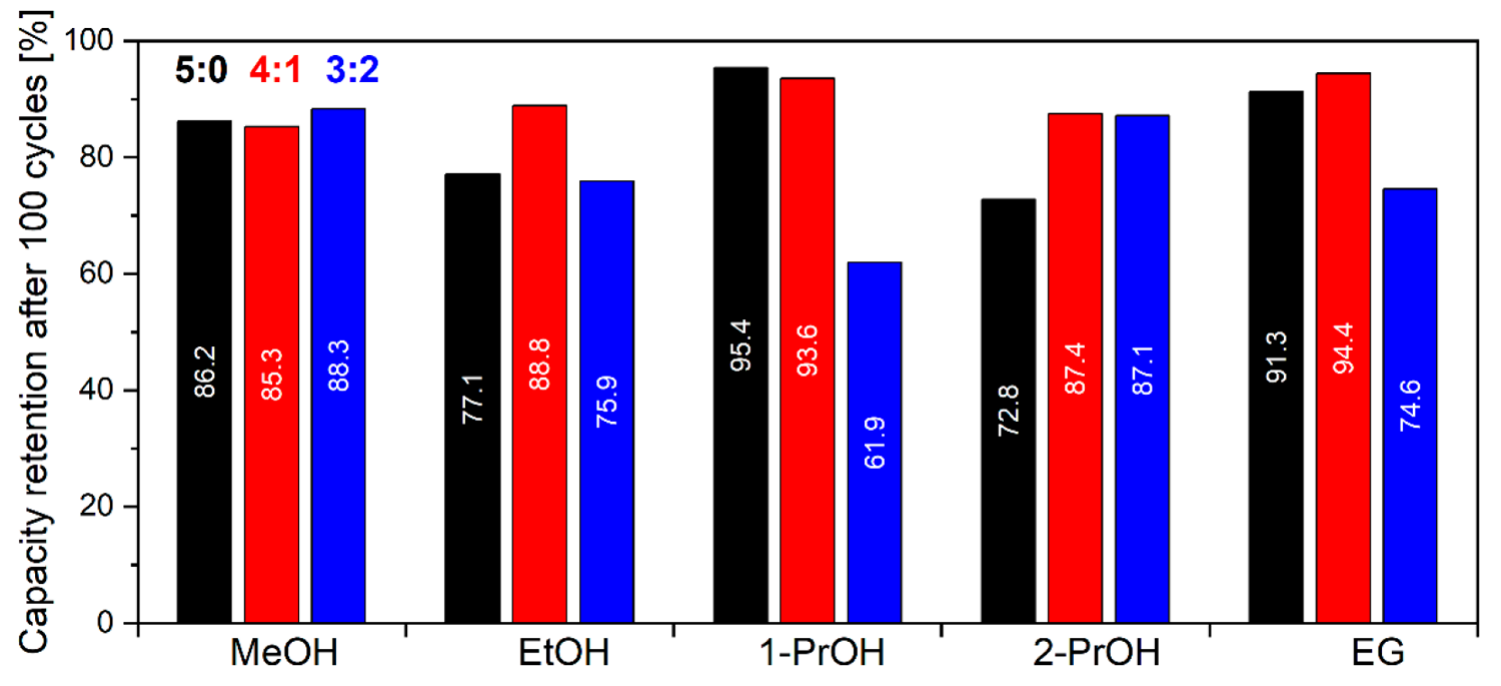
Capacity retention after 100 cycles of galvanostatic cycling performance of NTP prepared in different solvothermal media in 1 M Na_2_SO_4_ (aq) solution at 1C rate.

The specific discharge capacity, capacity retention, and Coulombic efficiency (CE) of NTP electrodes were investigated by means of galvanostatic charge/discharge (GCD) cycling within the potential window of −0.6 V to −0.9 V (Ag/AgCl) at 1C (1C = 0.133 A g^–1^) rate calculated based on the theoretical capacity of NTP (0.133 Ah g^–1^). Figures 10 and 11 represent 100 cycles of GCD cycling for different samples of NTP. The results show that the initial capacity and capacity retention are strongly correlated to NTP nanomorphology. Nanoparticles prepared from pure MeOH, 1-PrOH, and EG have higher initial capacities and less degradation, owing to their smoother surface and better homogeneity, high specific area, more active sites, and shorter diffusion paths which accelerate the insertion and extraction of Na^+^ ions. Capacity retention after 100 cycles was also calculated and represented in Figure 12. Irregular morphology of samples obtained from EtOH and 2-PrOH leads to faster degradation. As was indicated previously,^30^ active material dissolution is the main degradation cause; therefore, more particle surface defects and irregularities lead to faster capacity decay. The samples obtained from pure 1-PrOH show the best capacity retention of 95%. This stability could probably be attributed to the largest particle size among pure alcohols (Figure 4). However, adding water to 1-PrOH increases the particle size but does not lead to better capacity retention. This indicates another effect that most likely the particle morphology also plays an important role once the optimal particle size (∼120 nm in our case) is achieved.

## Conclusions

In this study, we successfully prepared NaTi_2_(PO_4_)_3_ nanoparticles with controlled size and morphology from different synthesis media by a hydro(solvo)thermal method. The analyzed synthesis media were different alcohols (methanol, ethanol, 1-propanol, 2-propanol, and ethylene glycol) and their mixtures with water at volume ratios of 4:1, 3:2, 2:3, respectively. The powder X-ray diffraction and scanning electron microscopy analysis results showed that the hydro(solvo)thermal reaction medium has very strong effects on the nanoparticle shape, size, and phase purity. The progressive addition of water to the synthesis medium leads to larger and less pure NaTi_2_(PO_4_)_3_ nanoparticles with α-Ti(HPO_4_)_2_·H_2_O identified as the main impurity phase. It was identified that relative water activity in mixtures with different alcohols is the key parameter governing the hydro(solvo)thermal synthesis. A semiempirical Aerosol Inorganic–Organic Mixtures Functional groups Activity Coefficients (AIOMFAC) thermodynamic model was used to estimate the relative water activity in different alcohol mixtures. The modeling results corroborated experimental data showing that reaction media where relative water activity exceeds ∼0.65 at ambient conditions lead to either a significant amount of α-Ti(HPO_4_)_2_·H_2_O impurity or no NaTi_2_(PO_4_)_3_ phase at all. 1-Propanol deviates the most from ideal mixing and showing the highest water activities even at low water contents, whereas ethylene glycol was close to ideal mixing and showed the lowest relative water activities among the studied alcohols. Additionally, the effects of NaTi_2_(PO_4_)_3_ nanoparticle size and morphology on the electrochemical performance in aqueous electrolyte were analyzed. The results show that the optimum size of particles for these applications is around 100 nm; however, the particle morphology also has a strong effect on capacity retention. Nanoparticles with irregular shapes and more surface defects display lower initial capacities and faster capacity fade. We show that hydro(solvo)thermal synthesis is a very versatile and powerful method to prepare NaTi_2_(PO_4_)_3_ nanoparticles for applications in aqueous Na-ion batteries. Careful selection and control of reaction medium and synthesis parameters in principle allow researchers to truly engineer nanoparticles with desired properties for diverse applications.

## Abbreviations

ASIBs: aqueous Na-ion batteries
NTP: NaTi_2_(PO_4_)_3_
THP: α-Ti(HPO_4_)_2_·H_2_O
MeOH: methanol
EtOH: ethanol
1-PrOH: 1-propanol
2-PrOH: 2-propanol
EG: ethylene glycol
XRD: powder X-ray diffraction
SEM: scanning electron microscope
TEM: transmission electron microscope
